# Phylogeography and genetic diversity of the widespread katydid *Ducetia japonica* (Thunberg, 1815) across China

**DOI:** 10.1002/ece3.7324

**Published:** 2021-03-08

**Authors:** Zhi‐Jun Zhou, Yun‐Xia Zhen, Bei Guan, Lan Ma, Wen‐Jing Wang

**Affiliations:** ^1^ College of Life Science Institute of Life Science and Green Development Hebei University Baoding China

**Keywords:** *Ducetia japonica*, ecological niche modeling, genetic diversity, microsatellites loci, mitochondrial COI‐5P, phylogeography

## Abstract

Habitat fragmentation can lower migration rates and genetic connectivity among remaining populations of native species. *Ducetia japonica* is one of the most widespread katydids in China, but little is known about its genetic structure and phylogeographic distribution. We combined the five‐prime region of cytochrome c oxidase subunit I (COI‐5P), 11 newly developed microsatellite loci coupled with an ecological niche model (ENM) to examine the genetic diversity and population structure of *D. japonica* in China and beyond to Laos and Singapore. Both Bayesian inference (BI) and haplotype network methods revealed six mitochondrial COI‐5P lineages. The distribution of COI‐5P haplotypes may not demonstrate significant phylogeographic structure (*N*_ST_ > *G*_ST_, *p* > .05). The STRUCTURE analysis based on microsatellite data also revealed six genetic clusters, but discordant with those obtained from COI‐5P haplotypes. For both COI‐5P and microsatellite data, Mantel tests revealed a significant positive correlation between geographic and genetic distances in mainland China. Bayesian skyline plot (BSP) analyses indicated that the population size of *D. japonica's* three major mitochondrial COI‐5P lineages were seemingly not affected by last glacial maximum (LGM, 0.015–0.025 Mya). The ecological niche models showed that the current distribution of *D. japonica* was similar to the species’ distribution during the LGM period and only slightly extended in northern China. Further phylogeographic studies based on more extensive sampling are needed to identify specific locations of glacial refugia in northern China.

## Graphical abstract

We investigated genetic diversity and population structure of D. japonica in China using the DNA barcoding fragment (COI‐5P) and 11 newly developed microsatellite loci. Three major lineages were nearly at a stable population size during LGM, and it does not appear that any lineages were adversely affected by climatic changes.

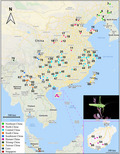

## 1 INTRODUCTION

Phylogeography focuses on the principles and processes underlying the geographic distributions of genetic lineages within single species (Avise, 2009). The population genetic structure and phylogeographic distribution of extant species are largely affected by various factors, such as geographic barriers (Hirao et al., 2015; Ye et al., 2016), ecological differences (Katz et al., 2018), and historical processes (Campos et al., 2013; Hewitt, 2000), as well as the dispersal potential of species (Francoso et al., 2016; Portnoy et al., 2015). Characterizing population structure and dispersal is also critical to understanding population dynamics (Levy et al., 2016). Geographic barriers play an important role in reducing gene flow and promoting genetic differentiation of extant species (Zhang et al., 2017). Higher dispersal propensity typically results in more gene flow and thus lower genetic structure between populations (Chamberland et al., 2020). Suitable environments with relaxed selective pressure can accommodate more genotypes and the population possesses higher genetic diversity (Jin & Liu, 2008). Habitat loss and fragmentation can lower migration rates and genetic connectivity among remaining populations of native species (Vandergast et al., 2007). Therefore, it is crucial to characterize the genetic lineages that may reflect species’ adaptive potential.

To date, phylogeographic analyses have been conducted in many insects (Kindler et al., 2012; Li et al., 2019; Matenaar et al., 2018; Villalta et al., 2018). Most widely distributed species subdivided into distinct evolutionary lineages or cryptic species (Barry et al., 2013; Yang et al., 2016). In contrast, high dispersal potential, human transportation, or host dissemination can lead to the population homogeneity of few widely distributed species (Li et al., 2019; Loureiro et al., 2020; Zhang et al., 2017).

Climatic oscillations during the Quaternary Period had a strong effect on the genetic diversity and distribution of extant species. During the Pleistocene glaciations, Europe and most regions of North America were covered with ice sheets. In the same period, there were no large and continuous ice sheets in China, except in some high mountains. The Hengduan Mountains are the most important refugial region in southwest China (Li et al., 2011; Liu et al., 2012; Meng et al., 2015; Yang et al., 2008; Zhang, 2002). Recent phylogeographic studies indicated that extant species’ genetic patterns at large scales are often shaped by past climate‐driven range dynamics. Most species retracted in small ice‐free refugial areas in the temperate zone of the northern hemisphere during the last glacial maximum (LGM, 0.015–0.025 Mya), and following a geographic expansion during the post‐LGM period (Yang et al., 2016). In southwest China, mountains form a series of parallel alpine areas over 5,000 m above sea level, and altitude drop between mountaintops and valleys exceeds 2000 m. The complex topography of southwest China led to long‐term geographic isolation. Several widely distributed insects exhibit high genetic variability and strong phylogeographic structure in China (Li et al., 2019; Yang et al., 2016).

Multiple molecular markers (e.g., single‐copy nuclear gene sequences, internal transcribed spacer, single nucleotide polymorphism, microsatellite loci, mitochondrial and chloroplast DNA) have been used for phylogeographic studies (Card et al., 2016; Garrick et al., 2015; Wang, Yang, Lu, Zhou, & Wu, 2017). Since single locus datasets can fail to adequately represent the overall and real lineage history of the species, increasingly more phylogeographic studies, therefore, have combined multiple molecular markers (Lu et al., 2016; Myers et al., 2013). Over the past two decades, phylogeographic datasets have become progressively larger in size (Garrick et al., 2015). The five‐prime region of cytochrome c oxidase subunit I (COI‐5P) has been widely used for distinguishing species, revealing cryptic species, and phylogeographic study (Lait & Hebert, 2018; Li et al., 2019; Mari‐Mena et al., 2016). The use of mitochondrial DNA has decreased considerably over the years, but still continues to represent an important component of the phylogeographic toolbox (Garrick et al., 2015). Microsatellites may provide higher resolution of differentiation than mitochondrial DNA, owing to their higher mutation rates. Thus, microsatellites are usually expected to be more informative in describing the consequences of the most recent population events (Royo et al., 2007).

While phylogeographic structure of many vertebrates has been examined in China (Dai et al., 2011; Dufresnes et al., 2016; Qiu et al., 2016), insects have received much less attention despite their central ecological roles (Jiang et al., 2016; Li et al., 2019). A better understanding of current patterns of genetic structure and geographic distributions of insects across China requires more phylogeographic studies. Few phylogeographic studies have reassessed the distribution of orthopteran insects combining mitochondrial and nuclear genetic markers on a broad geographic scale (Alfaro et al., 2018; Allegrucci et al., 2005, 2014; Ma et al., 2012). The *Ducetia japonica* group originated somewhere in East Asia and spread from there into the North (Japan), West (India), and South (Australia) (Heller et al., 2017). In China, *D. japonica* group encompasses *D. japonica* and *D. strelkovi* (Kang et al., 2014), which are mainly distinguished by the venation of forewings and, especially, by a long ventral ridge of the male cerci (Heller et al., 2017). A recent study based on acoustic data and stridulatory file variation suggested that *D. japonica* is a complex of several distinct species (Heller et al., 2017). *D. japonica* is a common katydid on *Ilex chinensis* in urban parks. The juveniles of *D. japonica* regularly visit and consume the pollinia and anther caps of *Habenaria sagittifera* (Suetsugu & Tanaka, 2014). *D. japonica* is widely distributed in China, which represents an ideal model system for the study of orthopteran population structuring on a broad geographic scale. To our knowledge, very little is known about the phylogeographic structure of *D. japonica* and other broad‐winged katydids. Herein, mitochondrial COI‐5P and 11 microsatellite loci were used together to study *D. japonica* genetic diversity and phylogeographic structure based on range‐wide sampling in China. Our aims are to (a) determine the phylogeographic structure and possible influential factors; (b) study the demographic history and gene flow among populations; and (c) identify the locations of putative refugia.

## 2 MATERIALS AND METHODS

### 2.1 Samples

**FIGURE 1:**
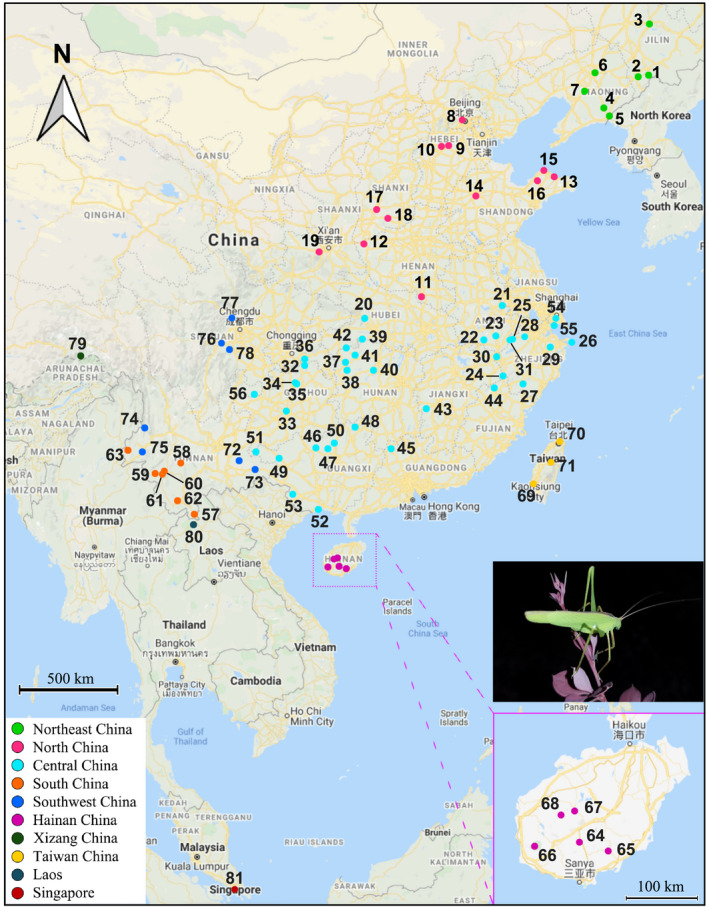
Geographic distribution of 81 *Ducetia japonica* populations. The numbers indicate the sampling localities specified in Table S1. The different colors represent the 10 population groups according to the zoogeographic divisions of China and potential geographic barriers

In total, 796 individuals were collected from 81 populations of *D. japonica* (Figure 1, Table S1). We collected from 1 to 60 individuals from each sampling site and identified them based on morphology (Kang et al., 2014). Specimens were preserved in absolute ethanol and stored at −20℃ until DNA extraction.

These populations were initially defined as 10 groups according to the zoogeographic divisions of China (Zhang, 2002) and potential geographic barriers: Northeast China (NEC, pops. 1–7), North China (NC, pops. 8–19), Central China (CC, pops. 20–56), South China (SC, pops. 57–63), Hainan China (HNC, pops. 64–68), Taiwan China (TWC, pops. 69–71), Southwest China (SWC, pops. 72–78), Xizang China (XZC, pop. 79), and locations outside China: Laos (pop. 80), and Singapore (pop. 81). To reduce the small sample bias, those populations with sample size less than 5 individuals were excluded from follow‐up population comparisons.

### 2.2 Mitochondrial COI‐5P sequencing and microsatellite genotyping

Genomic DNA was extracted from muscle tissue of each adult individual with the TIANamp Genomic DNA Kit (Tiangen, Beijing) according to the manufacturer's protocol. PCR was performed using Premix Taq™ (Takara, Beijing). Mitochondrial COI‐5P was amplified and sequenced using the universal primers LCO1490/HCO2198 (Folmer et al., 1994) or COBU/COBL (Pan et al., 2006).

We developed microsatellite loci specifically for *D. japonica* via 454 sequencing. Based on the initial analysis of amplification success rate and polymorphism, 11 microsatellite loci were amplified using fluorescent‐labeled primers (Table 1). The PCR system contained 7.5 μl 2 × Premix Taq™ (Takara Bio), 1.5 μl each primer (10 μM), 1 μl genomic DNA (~50 ng), and deionized water up to 15 μl. The amplification protocol of microsatellite loci included an initial denaturation at 94°C for 3 min, followed by 34 cycles of denaturation at 94°C for 30 s, annealing at 53–62°C (Table 1) for 40 s, extension at 72°C for 45 s, and a final extension at 72°C for 9 min. The amplification products were subjected to electrophoresis in a 2% agarose gel in TAE buffer to check whether the amplification reactions were successful. Amplified products were run on an ABI 3,730 XL Genetic Analyzer (Genewiz, China). Each reaction received 0.5 μl PCR products, 0.5 μl GeneScan™ 500 LIZ^®^ dye Size Standard (Thermo Fisher Scientific), and 9 μl Hi‐DiTM formamide (Thermo Fisher Scientific). Microsatellite alleles were genotyped by size using GeneMapper 4.0 (ABI) and checked manually to reduce scoring error.

**TABLE 1:** Primers used for microsatellite loci amplification

Locus	GenBank accession	Sequences (5′→3′)	Repeat motif	*T*_m_ (℃)
IV1EB	MN088333	F: CTGAGTGTTTGCGACGTTGT R: GAAGTACTGCGCGTGTGTGT	(CA)23	60–62
IB7C3	MN088334	F: CCGATTCCTGGAAAAGTTGA R: TCGCTGACGGTGTAAGAATG	(TCT)15	58–60
JOOO4	MN088336	F: TACCATTGCCTTTGCTCCTC R: TAGCACGCCTGACTTGAAAA	(TGT)28	55–58
IAK5J	MN088338	F: GGGCGTCTACAAAGAATTTCC R: GGAGCCACACTCTCAGGAAG	(GAGT)9	58–60
IIM2H	MN088339	F: TGGCCAGATCTACCATCACA R: CAATCAATCCGGGAAAAATG	(AGAC)9	55–58
ION7O	MN088340	F: GCAATTTGTGAATGCAAACG R: GCAATCTATGCCAACGACCT	(TATG)13	55–58
IXETP	MN088341	F: CGGAAGCGGTTAGATGTGTT R: GGAAAGGAAAAAGAAAGAAAGAA A	(TCTT)10	53–55
H87V5	MN088343	F: AAACCAGCTCTAGGCCTTCC R: GTCACGTCATCGTCATCGTC	(TAA)24	58
JM9VE	MN088344	F: ATGTGGGGGAAACATTTCAA R: AAAACAAAAATGGGAACATCC	(ATAG)17	55
JLV1A	MN088345	F: CTTTAGTTCACGGGGTCACG R: CCACACAGGAATTCTCAGCA	(AG)22	58
H49BE	MN088347	F: AAACCAGCTCTAGGCCTTCC R: AACGTCATCGTCATCGTCAC	(TAA)25	58–60

Abbreviations: *T*_m_, PCR annealing temperature.

### 2.3 Data analysis

#### 2.3.1 Genetic diversity and genetic differentiation

Mitochondrial COI‐5P genetic diversity indices, including the number of haplotypes (N), haplotype diversity (h), and nucleotide diversity (π), were calculated using DnaSP 5.10 (Librado & Rozas, 2009). For microsatellite data, the null allele frequency at each locus was calculated using GENEPOP 4.7 (Rousset, 2008). GenAlEx 6.5 (Peakall & Smouse, 2012) was applied to calculate the number of alleles (N_A_), observed heterozygosity (H_O_), expected heterozygosity (H_E_), and fixation index (F_IS_) for each locus and population. Polymorphism information content (PIC) was calculated in CERVUS v3.0. Genotypic linkage disequilibrium (LD) among all pairs of loci across all populations and Hardy–Weinberg equilibrium (HWE) tests for per locus and over all loci in each population were performed using GENEPOP 4.7 (Rousset, 2008). Myriads v1.1 was employed to detect heterozygote deficiency and heterozygote excess. Bonferroni corrections were used to adjust critical probability values for the above multiple tests (Rice, 1989).

Analysis of molecular variance (AMOVA) and population differentiation (*F*_ST_) were all implemented in Arlequin 3.5 (Excoffier & Lischer, 2010). AMOVAs were conducted to calculate the partitioning of genetic variation with 10,000 permutations. The pairwise genetic differences (*F*_ST_) range from 0 to 1, where “0” indicates complete panmixia and “1” suggests a lack of migration (Chabot et al., 2015). The conventional population *F*_ST_ comparisons were measured with 1,000 permutations at a significance level of 0.05. The spatial genetic pattern was examined by spatial analysis of molecular variance (SAMOVA) using SAMOVA 2.0 (Dupanloup et al., 2002). The SAMOVA was calculated for *K* = 2 to 15, and the initial condition was set to 100 with 10,000 iterations. The point at which the *F*_CT_ curve begins to plateau defines the final configuration of groups (*K*) (Heuertz et al., 2004).

#### 2.3.2 Phylogenetic network and divergence times based on mitochondrial COI‐5P sequences

Mitochondrial COI‐5P sequences were edited with Seqman and EditSeq (DNASTAR, Lasergene 7.1) and aligned with MUSCLE (Edgar, 2004) as implemented in MEGA 7 (Kumar et al., 2016). Haplotypes were identified with DnaSP v.5.10 (Librado & Rozas, 2009). Phylogenetic relationships among haplotypes were analyzed with both Bayesian inference (BI) and median‐joining (MJ) network methods. A BI phylogenetic tree was reconstructed using MrBayes 3.1.2 (Ronquist & Huelsenbeck, 2003) with HKY + G + I model, which was selected using PhyloSuite (Zhang et al., 2020). We performed two replicate analyses of 50 million generations each and sampled every 1,000 generations. *Kuwayamaea brachyptera* (accession number HQ609356) was used as out‐group. Stationarity was considered to be reached when the average standard deviation of split frequencies was below 0.01. The initial 25% of trees were discarded as burn‐in, and the remaining trees were used to construct the Bayesian majority‐rule consensus tree. Haplotype networks were derived from PopArt 1.7 using MJ algorithm (Leigh & Bryant, 2015). PERMUT 2.0 (Pons & Petit, 1996) was applied to test if the distribution of COI‐5P haplotypes demonstrated significant phylogeographic structure, the estimate of genetic differentiation for phylogenetically ordered alleles (*N*_ST_) significantly higher than population differentiation (*G*_ST_). As PERMUT software needed at least three individuals per population, we excluded the populations with sample size less than 5 individuals.

Divergence times were estimated using COI‐5P haplotype dataset with BEAST v1.10.4 (Suchard et al., 2018). Because no fossil record was available, we assumed a divergence rate of 3.54% per million years for mitochondrial COI‐5P (twice the substitution rate along one species/lineage), as previously estimated across insects for this locus (Papadopoulou et al., 2010). The divergence rate is twice the substitution rate along one species/lineage (Bensch et al., 2013). Clock.rate parameter was set as a normal prior with an initial value = 0.0177, mean = 0.0177, and *SD* = 0.004. The BEAST analysis was run 100 million generations with HKY + G + I model, strict molecular clock, random starting tree, the Yule process, and sampling every 10,000 steps. The first 10% of the trees were discarded as burn‐in. The maximum clade credibility tree was produced in TreeAnnotator v1.10.4.

#### 2.3.3 Demographic history based on mitochondrial COI‐5P sequences

To trace the demographic changes of each population and three major COI‐5P lineages of *D. japonica*, both neutrality test and mismatch distribution analyses were conducted in Arlequin 3.5 (Excoffier & Lischer, 2010). Both neutrality tests, Tajima's *D* and Fu's *Fs*, were used to evaluate historical demographic expansion with 1,000 simulated samples. Neutrality indices, Tajima's *D* and Fu's *Fs*, are widely used in molecular analysis to detect whether populations are under recent expansion. Tajima's *D* test (Tajima, 1989) is derived from mutation (segregating sites) frequencies, while the Fu's *Fs* test (Fu, 1997) is derived from haplotype distribution (Ramirez‐Soriano et al., 2008). Both Tajima's *D* and Fu's *Fs* values are expected to be near zero if population sizes have been stable. Significant negative values represent the population has undergone recent expansion, and positive values represent the population experienced a bottleneck (Fu, 1997; Tajima, 1989). Mismatch distribution for each population and three major COI‐5P lineages were conducted for the sum of squared deviations (SSD) and Harpending's raggedness index (HRI) (Excoffier, 2004; Harpending, 1994; Rogers & Harpending, 1992) under sudden expansion model by 100 bootstrap replications. This analysis quantifies smoothness of the observed mismatch distribution, and a nonsignificant result indicates population growth (Harpending, 1994).

Bayesian skyline plots (BSPs) were used to evaluate the population size dynamics of *D. japonica* three major mitochondrial COI‐5P lineages over time using BEAST v1.10.4 (Suchard et al., 2018). We focus on three major COI‐5P lineages (I, II, and III) because other lineages (IV, V, and VI) only contain 1 or 2 haplotypes. HKY + G + I model was determined to be the best substitution model using PhyloSuite (Zhang et al., 2020). Analyses were run using a strict molecular clock, assuming a divergence rate of 3.54% per million years (Clock.rate parameter was set as a normal prior with an initial value = 0.0177, mean = 0.0177, and *SD* = 0.004) (Papadopoulou et al., 2010) and generation time of 1 year. Two independent analyses were run 500 million generations and sampling every 50,000 steps (lineage I), and 200 million generations and sampling every 20,000 steps (lineage II and lineage III). Tracer v1.7.1 (Rambaut et al., 2018) was used to assess convergence (all ESS parameters were > 200) and visualization of median and 95% highest posterior probability density intervals (HPD).

#### 2.3.4 Population structure based on microsatellite data

Bayesian clustering analysis was implemented in Structure 2.3.4 (Pritchard et al., 2000) without prior structure information. All the possibilities were considered by dividing 81 populations into 10 groups, so we think the optimal number of clusters (K) will not exceed 15. Twenty runs for K = 1 to 15 were analyzed under the admixture model, correlated allele frequencies, and a burn‐in of 250,000 followed by 1,000,000 Markov chain Monte Carlo (MCMC) iterations. Structure Harvester 0.6.93 (Earl & Vonholdt, 2012) was applied to choose the optimal K‐value based on the Delta *K* method. The 20 replicates for the chosen *K*‐value were merged using CLUMPP 1.1.2 (Jakobsson & Rosenberg, 2007), and the final plots were generated using DISTRUCT 1.1 (Rosenberg, 2004).

#### 2.3.5 Correlation between genetic and geographic distance

To test for isolation by distance, Mantel tests were implemented with 1,000 permutations in Arlequin 3.5 to estimate correlation between genetic differentiation (*F*_ST_) and the shortest geographic distance for both mitochondrial COI‐5P and microsatellite data. The Mantel tests were limited to the populations in mainland China, and excluded HNC (pops. 64–68), TWC (pops. 69–71), XZC (pop. 79), Laos (pop. 80), and Singapore (pop. 81), to avoid the influence of geographic barriers, such as Taiwan Strait, Qiongzhou Strait, and Tibetan Plateau. Geographic distances were calculated based on the latitude and longitude of sampling sites.

#### 2.3.6 Current and LGM ecological niche models

The potential distribution of *D. japonica* was predicted using Maxent 3.3.3 (Phillips et al., 2006). The ecological layers for the current (1960–1990) and LGM dataset (Community Climate System Model, CCSM) were downloaded from WorldClim (http://www.worldclim.org) at 2.5 arc‐min resolution. In addition to our sampling sites, 41 species’ distribution records of *D. japonica* were obtained from the literature (Kang et al., 2014). We first constructed an ecological niche modeling (ENM) for current climate conditions using all environmental variables. Model validation was performed with 20 replicate runs using default convergence threshold with 10,000 maximum iterations and 25% of the sites for model training. The importance of environmental variables was determined by percentage contribution. Then, one of the highly correlated variables (correlation coefficient of |*r*| ≥ 0.7) with low‐explanatory power was removed based on its relative contribution to the Maxent model. Finally, 8 environmental variables were used to predict distribution areas of *D. japonica* in China: annual mean temperature (bio1), mean diurnal range (bio2), isothermality (bio3), temperature seasonality (bio4), precipitation of wettest month (bio13), precipitation of driest month (bio14), precipitation seasonality (bio15), and precipitation of coldest quarter (bio19). Model validation was performed using the same settings. The accuracy of each model prediction was evaluated using AUC scores, where range from 0.5 (randomness) to 1 (exact match), and above 0.7 and 0.9 indicates good and excellent predictive power, respectively (Fielding & Bell, 1997; Ye et al., 2014).

## 3 RESULTS

### 3.1 Mitochondrial COI‐5P haplotype network, population genetic diversity, and demographic history

We obtained 796 mitochondrial COI‐5P sequences that were each 658 bp long. None of COI‐5P sequences exhibited characteristics of nuclear pseudogenes (frame shifts or premature stop codons). We obtained 159 COI‐5P haplotypes (herein designated as H1‐H159) based on 138 polymorphic sites. Among 159 COI‐5P haplotypes, 12 haplotypes (H2, H3, H9, H12, H13, H27, H61, H62, H63, H66, H83, and H118) were observed in more than one group, 22 haplotypes (H1, H22, H11, H21, H26, H68, H76, H40, H74, H65, H77, H41, H30, H28, H98, H105, H96, H107, H132, H125, H152, and H149) were observed in different populations within groups, and 125 haplotypes were private to a single population (87 haplotypes were unique to single individual) (Table S1). Haplotype H3 (*n* = 166) displayed the widest geographic distribution, which was seen among samples originating in 23 populations from 4 groups. Meanwhile, H3 has more descendant haplotypes which also suggest it is most likely to be the ancestral haplotype (Castelloe & Templeton, 1994). The second most frequent haplotype H2 (*n* = 90) was seen in 8 populations from NEC and NC groups. The third most frequent haplotype, H12, was shared by 50 samples and occurred in 3 populations from NC and CC regions.

**FIGURE 2:**
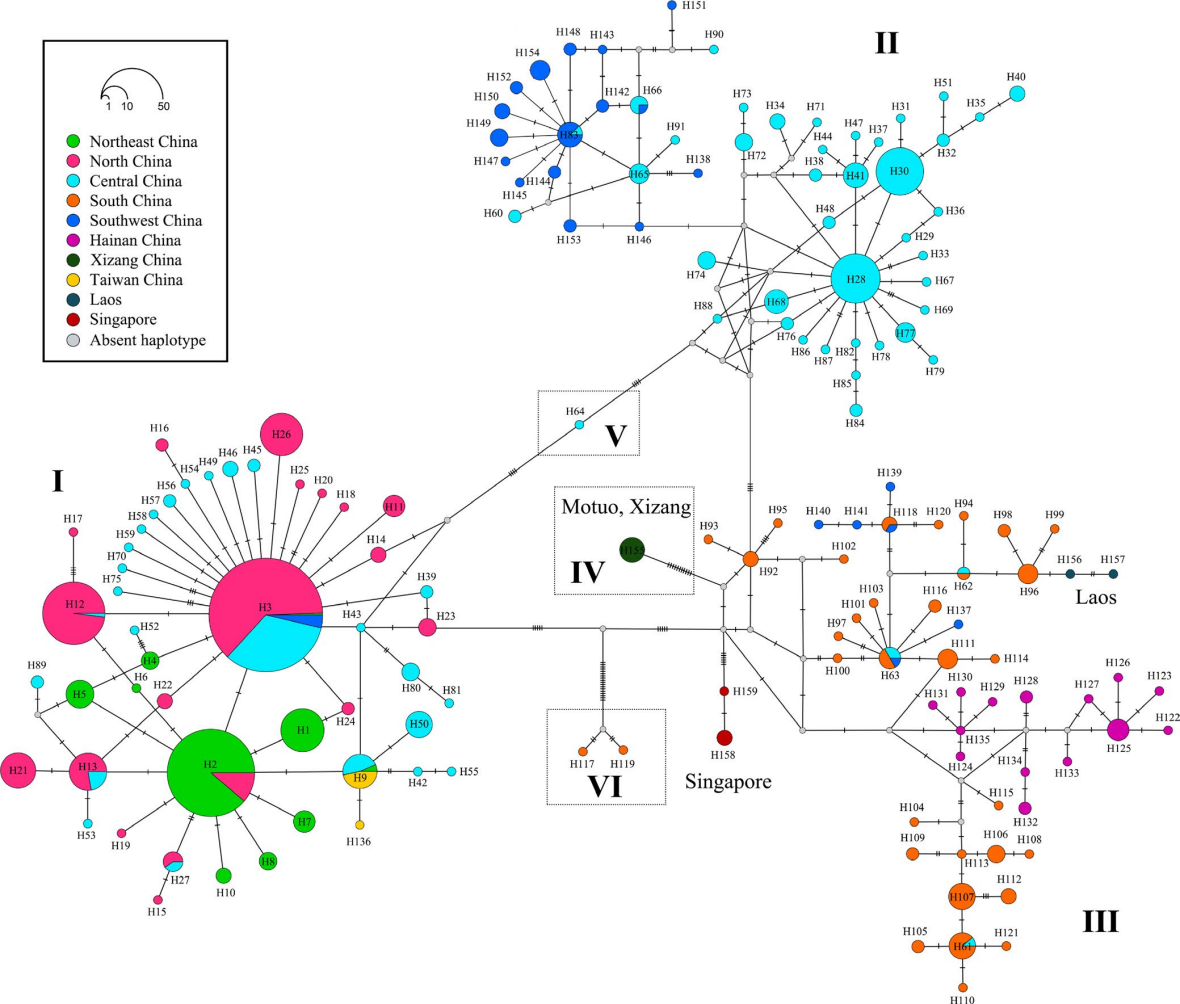
Median‐joining network showing the arrangement of the 159 mitochondrial COI‐5P haplotypes from *Ducetia japonica* samples. The haplotypes are shown by different colors based on 10 biogeographic regions. The size of circle is proportional to the number of sequences that make up specific haplotype, which ranged from 1 to 166. The small gray circles represent lost or unsampled haplotypes

BI analysis revealed six mitochondrial COI‐5P lineages (Figure S1). The COI‐5P haplotype network (Figure 2) displayed phylogeographic relationships consisted with those seen in the BI tree. Haplotype lineage I was composed of all members of the NEC, NC, and TWC groups, some of the members of the CC group, and a few members of the SWC group. H2 and H3 formed the core of the “star‐like” topology within haplotype lineage I. More surprisingly, dominant haplotype (H9) from Taiwan was shared with populations in NEC (LNSY) and CC (WHS, ZGTS) groups in which the maximum geographic distance was over 2000 km. Haplotype lineage II was composed of majority members of SWC and CC groups. H83 and H28 were the core of the “star‐like” topology within haplotype lineage II. Haplotype 64 from GZSY population was located at the junction of haplotype lineage I and lineage II. Haplotype lineage III was composed of all members of the HNC, SC, Singapore, and Laos groups, and a few members of the SWC and CC groups. H63 formed the core of the “star‐like” topology within haplotype lineage III. Two private haplotypes (H158 and H159) from Singapore separated from other haplotypes by more than 5 mutational steps. The private haplotype (H155) found in XZMT population formed haplotype lineage IV, which separated from other haplotypes by more than 10 mutational steps. Haplotype lineage VI was composed of two private haplotypes (H117 and H119) from single individuals of DDH population, and distantly related to the adjacent haplotype by more than 25 mutational steps. Within the CC group, the haplotypes from 6 populations (ZSX, ZQLF, GZCS, GDLZ, GXME, and HJS) were limited to haplotype lineage I, and 10 populations (WHS, ZJS, ZZS, ZJN, ZTMS, ZGTS, XSM, XTY, XSZ, and HMH) were assigned to either haplotype lineage I or II, and the remaining 20 populations were limited to haplotype lineage II. Both H2 and H12 were connected to H3 by one mutational step. Several singletons stemmed from haplotypes 2, 3, 28, 63, and 83 separated by a single substitution.

**FIGURE 3:**
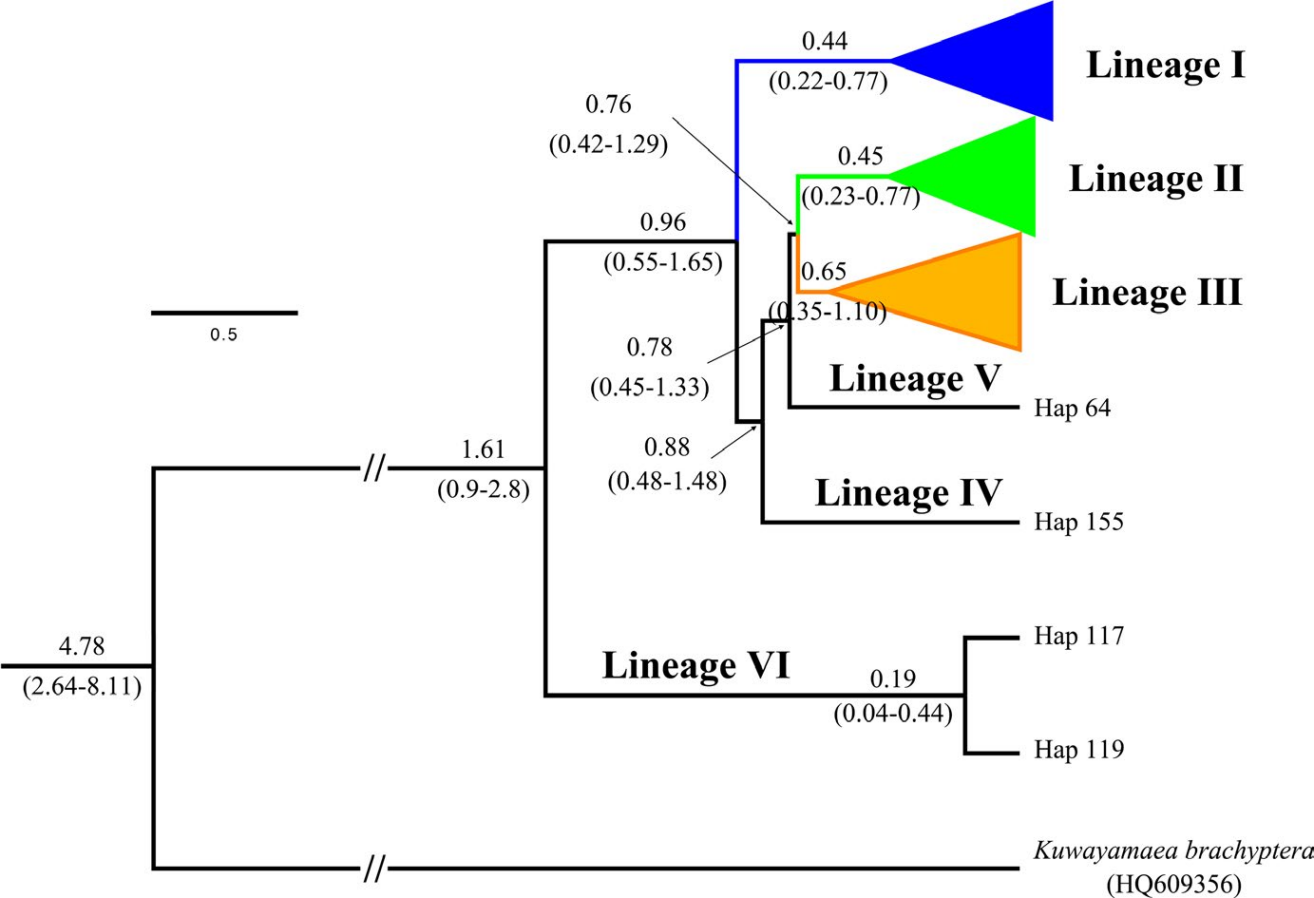
Divergence time estimation of *Ducetia japonica* using mitochondrial COI‐5P. Numbers above the lineages and in the brackets represent estimated divergence dates, and the 95% highest posterior density (HPD) of each node

Divergence time analysis retrieved the same topology with BI phylogenetic tree of *D. japonica* COI‐5P haplotypes. The primary divergence within *D. japonica* commenced 1.61 Mya (HPD: 0.9–2.8 Mya) when two haplotypes (H117 and H119) from DDH population split from the rest. Lineage I split from the other populations around 0.96 Mya (HPD: 0.55–1.65 Mya). The split between lineage II and lineage III was dated to 0.76 Mya (HPD: 0.42–1.29 Mya) (Figure 3).

The overall *D. japonica* mitochondrial COI‐5P haplotype (Hd) and nucleotide diversities (π) were 0.931 and 0.01391, respectively. The haplotype (Hd) and nucleotide diversities (π) of 4 populations (JLTH, JLJL, HJS, and XZMT) were zero for lack of variable sites, and the low diversity may be irrelevant to sample size (from 6 to 27 individuals). For the remaining populations, Hd ranged from 0.069 (WMAS) to 0.955 (CYA) and π ranged from 0.0001 (WMAS) to 0.0233 (DDH).

The neutrality tests indicated few signs of demographic events in most of the populations, except for SDYT and ZQLF, which showed significant negative values in both tests (Table 2). Four populations (JLTH, JLJL, HJS, and XZMT) consisted of a single haplotype each and could not be tested for neutrality.

**TABLE 2:** *Ducetia japonica* mitochondrial COI‐5P and microsatellite genetic diversity parameters

Population Code	Mitochondrial COI−5P								Microsatellites							
	*n*	Hap	Hd	Π	Tajima's D	Fu's FS	SSD	HRI	*n*	N_A_	N_E_	H_O_	H_E_	uH_E_	I	F_IS_
JLLJ	28	2	0.254	0.000	−0.019	0.448	0.275	0.307	28	11.545	5.926	0.597	0.719	0.732	1.824	0.144***
JLTH	20	1	—	—	—	—	—	—	20	11.091	6.405	0.611	0.750	0.769	1.896	0.201
JLJL	27	1	—	—	—	—	—	—	27	12.273	5.955	0.566	0.725	0.739	1.884	0.232
LNFC	28	2	0.071	0.000	−1.151	−1.155	0.000	0.740	28	12.545	5.119	0.538	0.707	0.720	1.784	0.222
LNDD	20	4	0.679	0.001	0.105	−0.304	0.022	0.168	20	9.273	4.478	0.573	0.692	0.710	1.623	0.161
LNSY	17	5	0.801	0.002	0.287	−0.688	0.052*	0.289*	16	9.182	4.946	0.522	0.684	0.707	1.659	0.233
BJHD	11	3	0.709	0.001	1.339	0.551	0.009	0.122	11	6.909	4.035	0.525	0.647	0.678	1.456	0.172
HBBD	60	6	0.451	0.002	−1.295	−0.826	0.264***	0.183	20	12.182	7.230	0.532	0.812	0.832	2.050	0.341
HBSP	10	4	0.711	0.003	−0.698	0.666	0.051	0.189	10	8.182	5.214	0.643	0.781	0.828	1.804	0.166
YLB	19	2	0.105	0.000	−1.511	0.021	0.016*	0.823	19	11.636	6.393	0.712	0.794	0.815	1.997	0.101
SDYT	24	3	0.163	0.000	−1.733*	−1.355*	0.006	0.579	24	9.000	4.530	0.491	0.677	0.691	1.591	0.252
SDJN	11	3	0.473	0.001	−0.290	−0.314	0.001	0.094	9	7.636	4.586	0.596	0.760	0.805	1.715	0.204
SDPL	19	5	0.526	0.001	−1.101	−1.386	0.002	0.070	19	11.727	5.536	0.650	0.778	0.799	1.945	0.152
SDZY	21	3	0.467	0.001	0.222	0.204	0.001	0.097	21	11.909	5.623	0.615	0.743	0.761	1.886	0.164
JSLF	13	5	0.782	0.003	−0.510	−0.504	0.018	0.071	13	10.091	6.202	0.624	0.786	0.818	1.941	0.197
JSQS	19	4	0.380	0.001	−0.644	−1.013	0.005	0.199	19	10.727	5.105	0.631	0.730	0.750	1.797	0.137
SSYL	36	3	0.475	0.001	−0.350	1.294	0.022	0.166	36	14.273	5.924	0.586	0.789	0.800	2.055	0.247
EDLL	6	2	0.333	0.001	−0.933	−0.003	0.003	0.222	—	—	—	—	—	—	—	—
WMAS	29	2	0.069	0.000	−1.149	−1.183**	0.000	0.748	24	12.000	6.503	0.606	0.741	0.757	1.897	0.153
WGNJ	7	4	0.810	0.006	0.722	1.092	0.150*	0.304	7	7.909	5.679	0.753	0.767	0.826	1.804	0.040
WHS	6	3	0.600	0.012	1.155	4.116	0.204	0.258	−	−	−	−	−	−	−	−
ZJS	10	5	0.867	0.010	0.174	2.333	0.647***	0.082	10	8.273	5.564	0.536	0.767	0.808	1.789	0.294
ZTMS	23	9	0.763	0.007	−0.559	0.264	0.046*	0.066	20	12.273	6.417	0.615	0.793	0.813	2.014	0.234
ZGTS	13	3	0.500	0.004	−1.917*	3.285	0.028	0.164	13	7.545	5.018	0.524	0.658	0.685	1.517	0.244
ZQLF	25	10	0.647	0.003	−2.254**	−4.709**	0.281***	0.061	—	—	—	—	—	—	—	—
XYS	9	3	0.417	0.001	−1.362	−1.081*	0.009	0.169	9	7.091	5.080	0.384	0.611	0.647	1.462	0.428
XGZ	6	2	0.333	0.002	−1.233	1.609	0.147*	0.667	6	6.000	4.884	0.591	0.669	0.730	1.477	0.137
XTY	8	4	0.750	0.015	0.780	4.117	0.225*	0.504***	8	6.818	4.920	0.523	0.692	0.738	1.550	0.238
JXJG	5	2	0.400	0.001	−0.817	0.090	0.007	0.200	5	5.636	4.383	0.818	0.736	0.818	1.536	−0.119
MWYS	5	2	0.400	0.002	−1.049	1.688	0.198	0.680	5	6.182	4.924	0.745	0.722	0.802	1.587	−0.042
GXHJ	7	4	0.810	0.002	−0.302	−1.217	0.028	0.213	7	6.000	4.172	0.429	0.647	0.696	1.421	0.347
GXME	5	2	0.400	0.001	−0.817	0.090	0.007	0.200	5	4.909	3.453	0.527	0.595	0.661	1.240	0.109
GXJZ	10	7	0.933	0.006	0.257	−1.217	0.031	0.069	9	7.455	5.335	0.495	0.652	0.690	1.557	0.202
GXLZ	5	2	0.400	0.001	−0.817	0.090	0.007	0.200	—	—	—	—	—	—	—	—
HMH	20	4	0.437	0.003	−1.811*	2.016	0.254***	0.444	20	13.636	7.352	0.795	0.840	0.862	2.199	0.049
HJS	6	1	—	—	—	—	—	—	6	5.909	4.573	0.682	0.688	0.751	1.487	−0.030
DML	16	12	0.950	0.009	−0.129	−3.495*	0.014	0.039	11	10.545	6.785	0.534	0.844	0.885	2.108	0.369
DGM	7	3	0.667	0.002	0.050	0.406	0.038	0.147	7	6.727	5.665	0.545	0.727	0.783	1.633	0.279
DLC	5	4	0.900	0.004	1.124	−0.445	0.017	0.070	5	5.545	4.453	0.482	0.739	0.823	1.538	0.358
DJH	26	12	0.914	0.008	0.616	−1.289	0.003	0.010	24	15.364	9.445	0.795	0.873	0.891	2.369	0.087
DDH	9	6	0.889	0.023	0.489	2.639	0.063	0.065	9	9.545	7.013	0.664	0.849	0.900	2.078	0.222
QWZS	8	6	0.893	0.003	−1.296	−2.676*	0.057	0.228	8	5.636	4.247	0.534	0.580	0.619	1.288	0.046
QDLS	6	5	0.933	0.005	0.050	−0.973	0.077	0.262	6	4.909	3.597	0.606	0.631	0.691	1.287	−0.010
QJFL	5	3	0.700	0.004	−0.668	1.090	0.185	0.590	—	—	—	—	—	—	—	—
CYA	12	9	0.955	0.007	−1.373	−2.685	0.050	0.161	12	10.273	6.702	0.636	0.810	0.845	1.999	0.226
CDJY	13	5	0.756	0.003	−0.990	−0.504	0.035	0.176	13	8.727	5.604	0.572	0.749	0.779	1.771	0.231
CEMS	15	6	0.829	0.012	1.780	3.451	0.868***	0.319	14	8.818	5.238	0.555	0.695	0.721	1.665	0.223
XZMT	8	1	—	—	—	—	—	—	7	6.273	4.670	0.571	0.701	0.755	1.524	0.217
Lineage I	507	48	0.838	0.003	−2.151***	−27.396***	0.001	0.061*								
Lineage II	173	54	0.931	0.005	−1.728**	−25.983***	0.006	0.015								
Lineage III	105	53	0.973	0.010	−1.177	−24.946***	0.002	0.007								

Abbreviations: *H*_E_, Expected heterozygosity; *F*, Fixation index; Hd, Haplotype diversity; HRI, Harpending's raggedness index; *N*_A_, No. of different alleles; *N*_E_, No. of effective alleles; Hap, No. of haplotypes; *n*, No. of samples; NA, Not applicable; *π*, Nucleotide diversity; *H*_O_, Observed heterozygosity; *I*, Shannon's information index; SSD, Sum of squared deviations; uHe, Unbiased expected heterozygosity.

* *p* < .05.

** *p* < .01.

*** *p* < .001.

**FIGURE 4:**
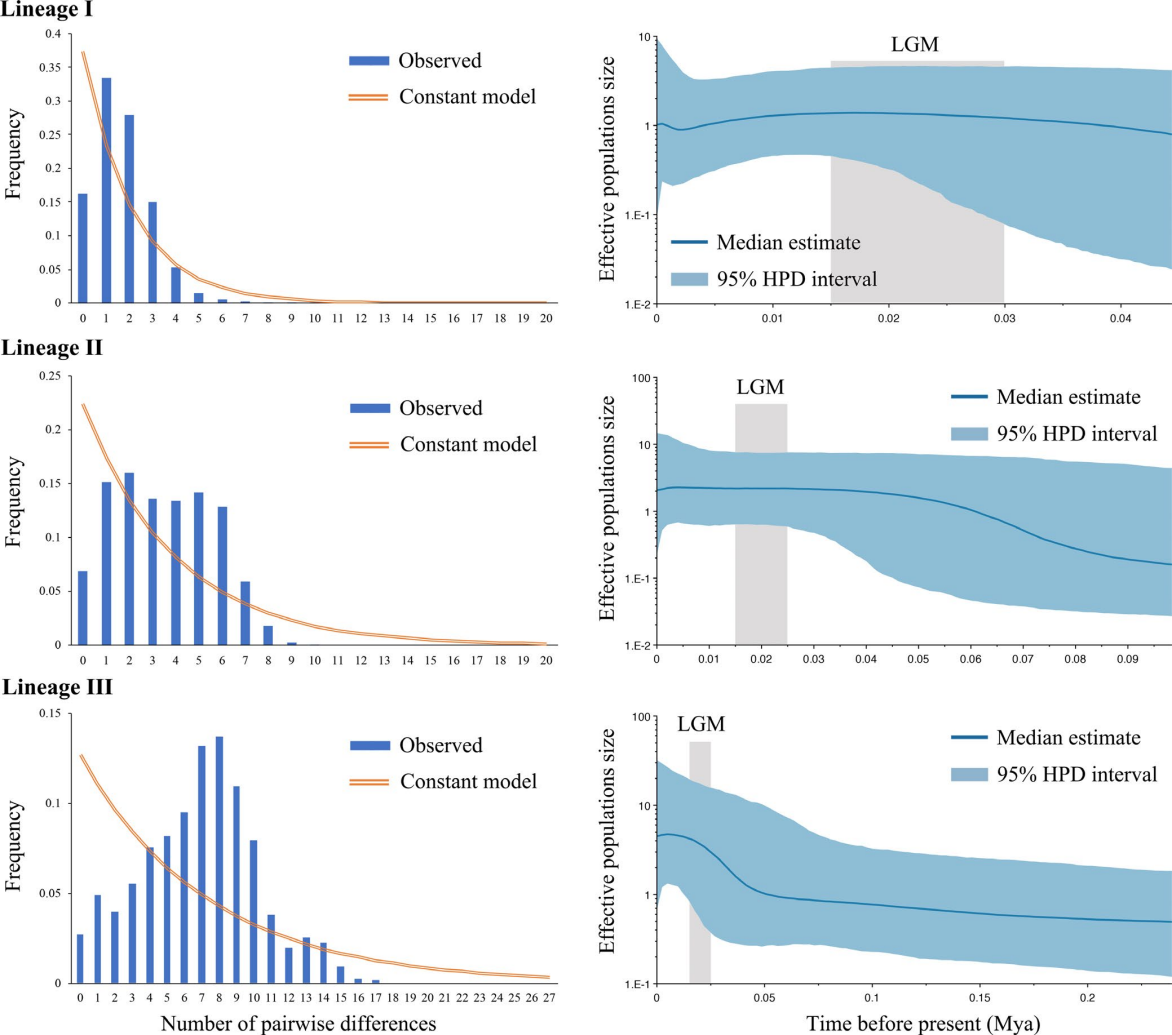
Mismatch distributions (left) and Bayesian skyline plots (right) for COI‐5P lineages I, II, and III of *Ducetia japonica*

Tajima's D test for lineage I (*p* < .001) and lineage II (*p* < .01) was significantly negative, whereas lineage III was negative but not significant. Fu's Fs test was significantly negative values (*p* < .001) for all lineages. Significantly negative values of Fu's Fs and Tajima's D tests support an excessive number of rare haplotypes as a result of a recent population expansion. The unimodal mismatch distribution pattern suggested that three lineages underwent recent expansions (Figure 4a). Significantly negative neutrality test values and unimodal mismatch distribution pattern suggested that the population fit the sudden expansion model.

According to the BSP results, lineage I showed a constant population size trend over time. Lineage II showed a relatively slight demographic expansion beginning around 0.09 Mya, and the effective population size remained stable beginning around 0.04 Mya. The effective population size of lineage III remained stable for a long period and then showed a demographic expansion beginning around 0.05 Mya, which lasted until recently. Moreover, it showed that none of the lineages was adversely affected by LGM (Figure 4b).

### 3.2 Microsatellite genotyping, population genetic diversity, and structure

Eleven microsatellite loci were genotyped for 610 *D. japonica* individuals from 43 populations. These microsatellite loci had different degrees of diversity, varying from 42 alleles (IIM2H and IB7C3) to 67 alleles (IAK5J) (Table 3). Significant heterozygote deficiency occurred in all microsatellite loci and most populations, except JXJG population. A total of 128 private alleles (restricted in a single population) were detected in 39 populations. Most populations have no more than private alleles, except DML (6), SSYL (7), DJH (7), JLLJ (10), LNFC (11), and WMAS (12). The number of effective alleles (N_E_) ranged from 7.516 (JM9VE) to 27.742 (IAK5J). Shannon's information index (I) values across loci ranged from 2.111 (IIM2H) to 3.615 (IAK5J). Observed heterozygosity (H_O_) ranged from 0.449 (IB7C3) to 0.712 (H87V5), and expected heterozygosity (H_E_) and unbiased expected heterozygosity (uHe) values ranged from 0.732 (IIM2H) to 0.964 (IAK5J) and 0.733 (IIM2H) to 0.965 (IAK5J), respectively. Fixation index (F_IS_) ranged from 0.253 (H87V5) to 0.494 (IB7C3). These ranges suggest differential mutation rates among loci.

**TABLE 3:** Genetic diversity at 11 microsatellite loci in 610 *Ducetia japonica* individuals

Locus	*n*	N_A_	N_E_	I	H_O_	H_E_	uH_E_	F_IS_
H87V5	607	60	21.209	3.364	0.712	0.953	0.954	0.253*
JLV1A	607	51	21.081	3.296	0.679	0.953	0.953	0.287*
JM9VE	603	53	7.516	2.753	0.614	0.867	0.868	0.292*
H49BE	610	62	14.758	3.289	0.662	0.932	0.933	0.290*
IIM2H	610	42	3.733	2.111	0.500	0.732	0.733	0.317*
IB7C3	610	42	8.872	2.653	0.449	0.887	0.888	0.494*
ION7O	610	45	17.623	3.252	0.564	0.943	0.944	0.402*
JOOO4	601	53	16.396	3.146	0.571	0.939	0.940	0.392*
IAK5J	610	67	27.742	3.615	0.634	0.964	0.965	0.342*
IXETP	608	52	14.273	3.093	0.558	0.930	0.931	0.400*
IV1EB	609	48	13.646	3.076	0.627	0.927	0.927	0.323*
Mean		52.273	15.168	3.059	0.597	0.912	0.912	

Abbreviations: *F*_IS_, Fixation index; *H*_E_, Expected heterozygosity; *H*_O_, Observed heterozygosity; *I*, Shannon's information index; *n*, Sample size; *N*_A_, No. of observed alleles; *N*_E_, No. of effective alleles; uHe, Unbiased expected heterozygosity.

* *p* < .001.

Mean N_A_ and N_E_ across all loci were highest in DJH (15.364 and 9.445) and lowest in the GXME population (4.909 and 3.453). The highest and lowest Shannon's information index (I) was presented in DJH (2.369) and GXME (1.24), respectively. The mean H_O_, H_E_, and uH_E_ across all loci were highest in JXJG (0.818), DJH (0.873), DDH (0.9), and lowest in XYS (0.384), QWZS (0.58), and QWZS (0.619), respectively.

**FIGURE 5:**
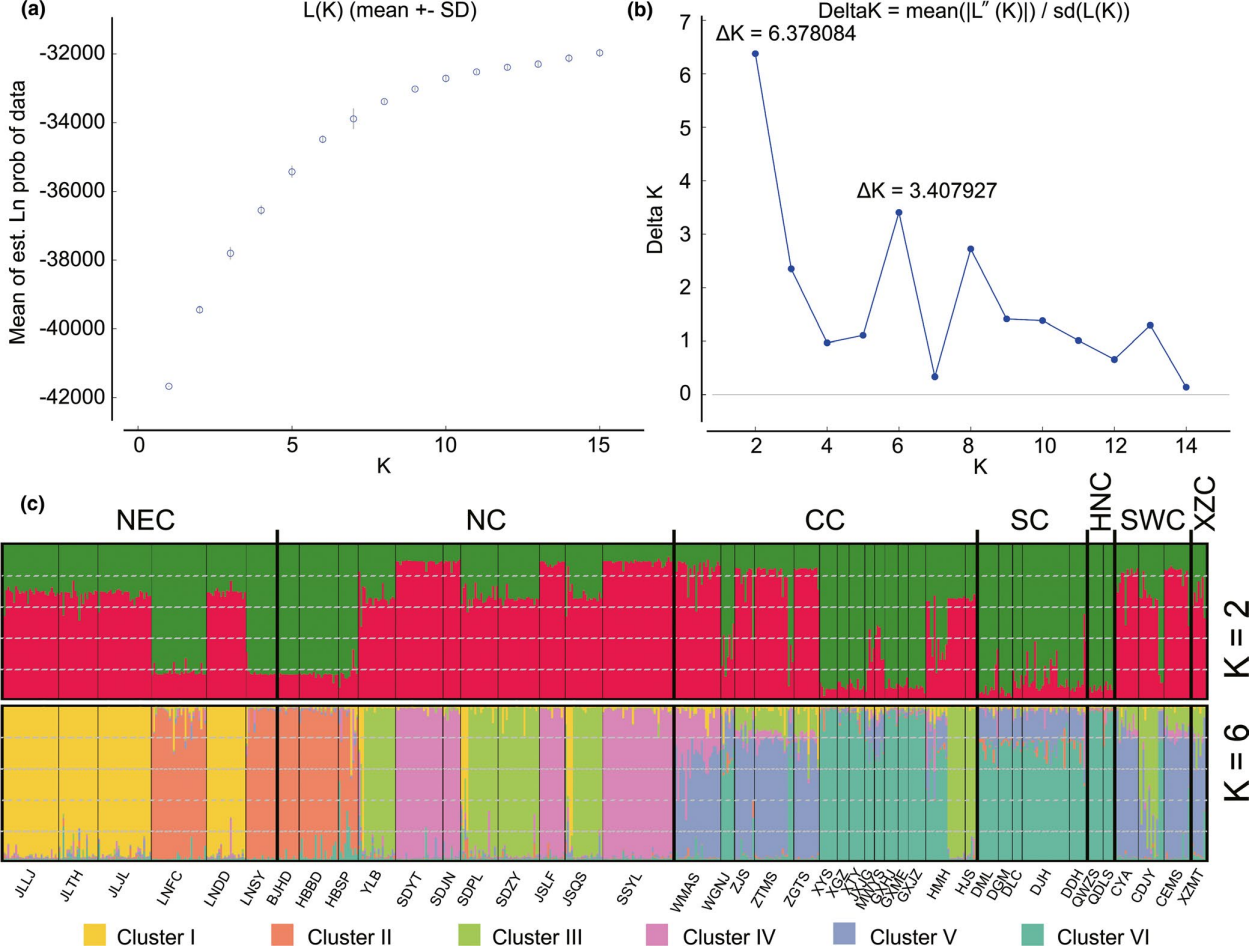
STRUCTURE clustering results based on microsatellite data of *Ducetia japonica*. (a) the posterior probability of the data given each K (20 replicates); (b) the distribution of ⊿*K* values; (c) Bayesian clustering results at *K* = 2 and 6 from the structure analysis

The STRUCTURE analysis based on microsatellite data indicated that the mean estimated logarithm of probability of the data, L (K) value increased gradually from *K* = 2–15 (Figure 5a). Plots of Δ*K* versus K showed multiple peaks at *K* = 2 (Δ*K* = 6.378), and *K* = 6 (Δ*K* = 3.408) (Figure 5b). At K = 2 (Figure 5c), the individuals of almost all populations were significantly assigned to two inferred clusters (*Q* > 0.90). STRUCTURE tends to detect the uppermost level of hierarchical structure, which may explain why *K* = 2 was frequently reported, despite our suspicions of additional underlying structure (Evanno et al., 2005; Levy et al., 2016). So, we also examined clustering based on the second highest Δ*K* = 3.408 occurred at *K* = 6. We found genetic similarities among populations from SC and HNC. The individuals collected from 6 populations (YLB, SDPL, JSQS, ZTMS, HMH, and CDJY) were assigned to two inferred clusters (*Q* > 0.80). Most individuals of SC, SWC, and XZC regions were inferred to be a mixture of two genetic components. Cluster I comprised samples of 3 populations in Jilin and LNDD population in Liaoning Province. Cluster II comprised samples of the remaining 2 populations in Liaoning, and BJHD population in Beijing and 2 populations in Hebei Province. Cluster III comprised samples of YLB population in Henan, SDPL and SDZY population in Shandong, JSQS population in Shanxi, HMH (partial individuals) and HJS population in Shanghai, and CDJY population in Sichuan Provinces. Cluster IV comprised samples of SDYT and SDJN population in Shandong, JSLF population in Shanxi, and SSYL population in Shaanxi Provinces. Cluster V comprised samples of WMAS population in Anhui, all populations in Zhejiang, CYA and CEMS populations in Sichuan Provinces, and XZMT population in Xizang. Cluster VI comprised samples of WGNJ population in Anhui, HMH (partial individuals) population in Shanghai, and all populations in Hunan, Jiangxi, Fujian, Guangxi, Yunnan and Hainan Provinces.

### 3.3 Genetic differentiation and Mantel test on isolation by geographic distance

In this study, the maximum K2P distance of COI‐5P sequences is 1.41%. The mean gene diversity (*H*_S_ = 0.548 ± 0.043) was significantly lower than total gene diversity (*H*_T_ = 0.961 ± 0.014). Although *N*_ST_ (0.448 ± 0.051) > *G*_ST_ (0.430 ± 0.042), but not significant (*p* >.05). Therefore, COI‐5P haplotype distribution may not demonstrate significant phylogeographic structure.

The SAMOVA based on mitochondrial COI‐5P dataset showed that F_CT_ value clearly increased from K = 2 to 4 and then slightly changed from *K* = 4 to 15. Four groups (*F*_CT_ = 0.735, *p* < .001) were delimited. Group I included all populations of NEC, NC, and TWC, and 12 populations (WHS, ZSX, ZZS, ZTMS, ZGTS, ZQLF, GZCS, XSM, XSZ, GDLZ, GXME, HMH, and HJS) of CC. Group II only included XZMT population from XZC alone. Group III included 23 populations (EDLL, WMAS, WGNJ, ZJS, ZJN, ZSMS, GZDZ, GZSY, GZKS, XYS, XGZ, XTY, XZJJ, JXJG, MWYS, GXHJ, GXLC, GXTL, GXRS, GXJZ, GXFC, GXLZ, and DZT) of CC and 4 populations (DQB, CYA, CDJY, and CEMS) of SWC. Group IV included all populations of SC, HNC, Laos, Singapore, and GZGY population of CC, and 3 populations (DWS, DNJ, and DBS) of SWC.

Results of the AMOVA test on mitochondrial COI‐5P and microsatellite data in different populations and regional groups are shown in Table 4. For mitochondrial COI‐5P, global AMOVA revealed that 21.54% genetic variation was found within populations, whereas 78.46% genetic variation was explained by differences among populations. Based on SAMOVA results, the most genetic variation was found among 4 groups (73.54%), followed by the genetic variance within populations (14.29%) and among populations within groups (12.17%). Regarding microsatellite data, global AMOVA revealed that 14.83% genetic variation was explained by differences among populations and up to 85.17% genetic variation was found within populations. Based on STRUCTURE clustering results, the most genetic variation was found within populations (84.09%), followed by the genetic variance among 6 groups (8.52%) and among populations within groups (7.40%). All fixation indices are significant (*p* < .001).

**TABLE 4:** Analysis of molecular variance (AMOVA) for *Ducetia japonica* samples based on mitochondrial COI−5P and microsatellite dataset

Source of variation	Mitochondrial COI−5P		Microsatellite	
	V%	*F*‐statistic	V%	*F*‐statistic
Global analysis				
Among population	78.46%	*F*_ST_ = 0.785*	14.83%	*F*_ST_ = 0.148*
Within population	21.54%		85.17%	
SAMOVA group (K = 4)				
Among groups	73.54%	*F*_CT_ = 0.735*		
Among populations within groups	12.17%	F_SC_ = 0.460*		
Within population	14.29%	*F*_ST_ = 0.857*		
STRUCTURE clusters (*K* = 6)				
Among groups			8.52%	*F*_CT_ = 0.085*
Among populations within groups			7.40%	*F*_SC_ = 0.081*
Within population			84.09%	*F*_ST_ = 0.159*

Abbreviations: V%, Percentage of variation.

* *p* < .001, 1,000 permutations.

In both mitochondrial COI‐5P and microsatellite data, population differentiation was significant (*p* < .05, 10,100 replicates) for the majority of pairwise comparisons. Pairwise *F*_ST_ between populations based on mitochondrial COI‐5P (Kimura‐2P) and microsatellite data (number of different alleles, *F*_ST_‐like) ranged from 0 to 1 and from 0.013 to 0.311, respectively (Table S2). There was lower *F*_ST_ among 4 populations (JLLJ, JLTH, JLJL, and LNFC) within NEC regions, and the *F*_ST_ values with the remaining 2 populations (LNDD and LNSY) were higher based on microsatellite data. Based on mitochondrial COI‐5P, lower *F*_ST_ occurs only among JLTH, JLJL, and LNFC, and the *F*_ST_ values exceeded 0.8 between them and JLLJ. Furthermore, lower *F*_ST_ often was observed between geographically adjacent populations, for example, HBBD and HBSP (microsatellite data: *F*_ST_ = 0.052 and mitochondrial COI‐5P: *F*_ST_ = 0.078), XYS and XGZ (microsatellite data: *F*_ST_ = 0.053 and mitochondrial COI‐5P: *F*_ST_ = 0.025), HMH and HJS (microsatellite data: *F*_ST_ = 0.060 and mitochondrial COI‐5P: *F*_ST_ = 0.036), and CYA and CDJY (microsatellite data: *F*_ST_ = 0.090 and mitochondrial COI‐5P: *F*_ST_ = 0.049). Our results indicated a low or moderate gene flow, as observed by the high and significant *F*_ST_ values. The Singapore lineage is much more divergent from the others; *F*_ST_ ranged from 0.429 to 0.997 for mitochondrial COI‐5P, and *F*_ST_ ranged from 0.117 to 0.364 for microsatellite data.

The geographic distance among populations ranged from 2.6 km (GZSY vs. GZKS) to 3,781.94 km (JLJL vs. DDH) in mainland China. Pairwise *F*_ST_ between the two nearest populations (GZSY and GZKS) was 0.23881 for microsatellites and 0.932 for COI‐5P. Mantel tests revealed a significant correlation between genetic differentiation and geographic distance for both mitochondrial COI‐5P (*r* = .452, *p* < .001) and microsatellites (*r* = .246, *p* < .001).

### 3.4 Distribution modeling

**FIGURE 6:**
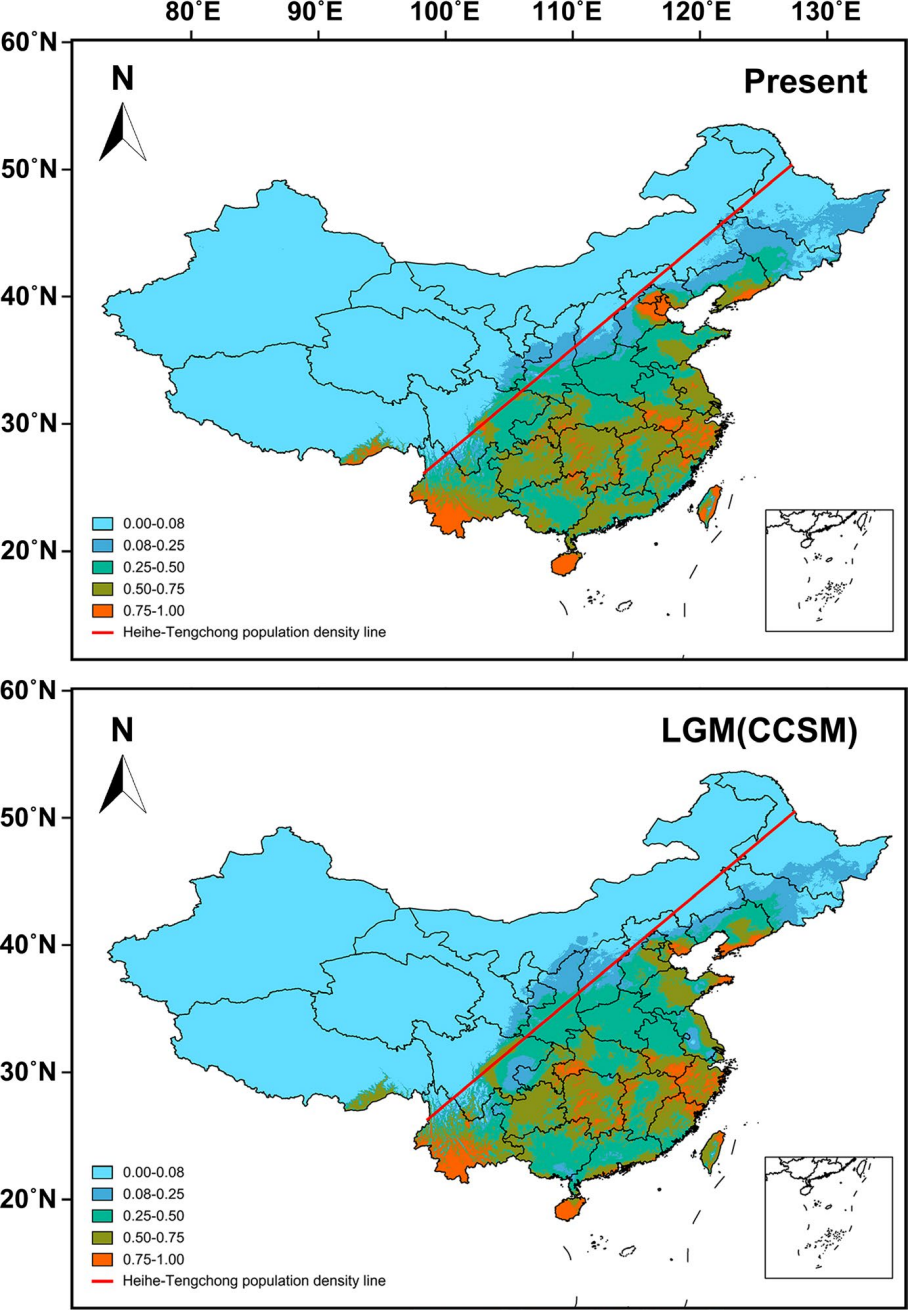
Predicted distribution area of *Ducetia japonica* under LGM and current climatic conditions

The average test AUC value for the replicate runs is 0.883 ± 0.042 for current and 0.871 ± 0.052 for LGM distributional predictions. These mean values showed that our models were distinct from random expectation. The predicted distribution range of *D. japonica* is mainly located in the east of Heihe–Tengchong population density line, especially Northern and Central China. The distribution predictions under current climatic conditions were largely congruent with the actual distribution of *D. japonica*. The predicted current distribution of *D. japonica* was similar to that of the LGM period and only slightly extended in Northern China (Figure 6). The predicted distribution with high suitability scores (>0.75) during LGM was slightly reduced in Northern China.

## 4 DISCUSSION

Our sampling scheme covered *D. japonica's* major geographic distribution in China and beyond to Laos and Singapore. The 159 COI‐5P haplotypes formed 6 haplotype lineages. Most haplotypes (125 of 159) were unique to a population, which also implies high level genetic differentiation among *D. japonica* populations. Several singletons stemmed from haplotype H2, H3, H28, H63, and H83 separated by a single substitution. This star‐like topology is often associated with recent demographic expansions (Alfaro et al., 2018). The random distribution of haplotypes implied the presence of relatively high gene flow among populations (Wang et al., 2017). SAMOVAs revealed four groups of populations in which three groups comprised the populations from Central China. No haplotypes were shared between XZMT and other populations. Haplotypes (H155) from XZMT population were connected with adjacent haplotype by more than 10 mutational steps, which may represent separate species, diverging lineages, or geographically isolated demes. SAMOVAs also showed that XZMT population formed a separate group. The STRUCTURE analysis based on microsatellite data also revealed 6 genetic clusters, but there were discordant with those obtained from COI‐5P haplotypes. XZMT population and multiple populations from Anhui, Zhejiang, and Sichuan were assigned to one cluster. The mito‐nuclear discordance may be the result of sex‐specific gene flow, secondary admixture, and/or incomplete lineage sorting (Kindler et al., 2012; Mari‐Mena et al., 2016; Rodrigues et al., 2014; Toews & Brelsford, 2012).

Morphologically, five species within the *D. japonica* group are very similar and differ mainly in song (Heller et al., 2017). *D. malayana* was described from Singapore based on song unit of calling song (Heller et al., 2017). The Singapore population had two unique haplotypes (H158 and H159), which connected with adjacent haplotype by more than 5 mutational steps. Two closely related haplotypes (H9 and H136) were found in three populations of Taiwan island. Dominant haplotype (H9) was shared with NEC and CC groups. The SAMOVAs also supported this situation.

The COI‐5P haplotype distribution may not demonstrate a phylogeographic structure (*N*_ST_ > *G*_ST_, *p* > .05). The high nucleotide diversity (*π* = 0.023) of DDH population can be explained by the existence of H117 and H119. High Hd and low *π* were observed in most populations, which probably resulted from rapid population growth of ancestral populations (Avise, 2000; Yu et al., 2014). The high genetic diversity might reflect a long evolutionary history and wide distribution of species (Li et al., 2019). Biogeographic barriers (such as rivers, mountains, and deserts) separate populations, impede gene flow, drive genetic differentiation, and eventually lead to allopatric speciation. The Hengduan Mountains are the most important refugial region in China (Liu et al., 2012).

*D. japonica* is a long‐winged species and one of the most widespread katydids found from Japan to Australia (~5,000 km) and Pakistan to Solomon Islands (~10,000 km) (Heller et al., 2017). Mantel tests revealed a significant positive correlation between genetic differentiation and geographic distance based on both mitochondrial COI‐5P and microsatellites. Population differentiation tends to be closely related to geographic distances (Wu et al., 2019). Dispersal ability has an important impact on genetic diversity of the species. With its long fore and hind wings, *D*. *japonica* group occurred on numerous islands, which is evidence to support its longer distance dispersal capabilities. The distribution on islands may be the result of different circumstances, such as the species’ arrival occurred when islands and mainland were not yet separated or accidental introduced by humans. Island populations were generally characterized as having lower genetic variation (Liu et al., 2020). Taiwan island was first isolated from mainland China by Taiwan Strait at ∼5 Ma. Hainan island was previously located near Guangxi and northern Vietnam during the early Cenozoic and formed approximately 2–2.5 Ma. *D. japonica* is a common katydid in urban parks of China and may be dispersed by trafficking in *I. chinensis*. Long‐distance, human‐assisted dispersal may have important implications for the genetic structure of many species (Dergousoff et al., 2016; Fresia et al., 2013; Ma et al., 2012; Woodin et al., 2014).

Meanwhile, significant *F*_ST_ values indicated that the genetic variation of *D. japonica* was highly structured. Our results revealed that there is a restricted gene flow among *D. japonica* populations. The complex topography, such as mountains and rivers, has acted as substantial barriers to gene flow within numerous species of insects distributed in East Asia (Liu et al., 2018). Significant negative values of Tajima's *D* and Fu's *Fs* may be the result of population expansion. Normally, the value of Fu's *Fs* is more sensitive than Tajima's *D* (Pilkington et al., 2008). The significant Fu's *Fs* values and star‐shaped haplotype networks are characteristic of species that have undergone a recent process of expansion or selection (Wares, 2010). Three major lineages of *D. japonica* were formed before the LGM. Lineage I showed a stable population size trend over time. Both lineage II (0.09 Mya) and lineage III (0.05 Mya) expansion may have occurred earlier than the LGM. Three major lineages were nearly at a stable population size during LGM, and it does not appear that any lineages were adversely affected by climatic changes. High levels of lineage diversity and signals of greater demographic stability suggest a lower impact of glaciations (Alfaro et al., 2018).

Many extant species persisted in small ice‐free refugial areas during LGM in the temperate zone of the northern hemisphere (Yang et al., 2016). As the average test AUC value was higher than 0.85 for both current and LGM climate conditions, we assumed that the models generated reliable predictions. Hence, we believe *D. japonica* has LGM refugia in North China. The predicted current distribution of *D. japonica* was similar to that of the LGM period and only slightly extended in northern China. This pattern could be because Southern China did not experience large‐scale glaciations during LGM, due to the blocking of cold winds by the Tibetan Plateau (Yang et al., 2016). Postglacial range expansion led to admixture between recolonizing populations. The distribution of *D. japonica* COI‐5P haplotypes might have resulted in the post‐LGM expansion from multiple refugia and secondary contact. The most likely scenarios suggest that current distribution of *D. japonica* originated from local recolonization from multiple refugia, rather than long‐distance migration. Heihe–Tengchong population density line is a well‐known demographic and economic‐graphical dividing line in China, which runs from Heihe in Heilongjiang Province to Tengchong in Yunnan Province. The distribution range of *D. japonica* is mainly located in the east of Heihe–Tengchong line, especially the Northern and Central China. Heihe–Tengchong line coincides with the 400 mm precipitation line, which suggests the effects of precipitation on *D. japonica* distribution. The population distribution and dynamics of plant‐feeding insects (e.g., *Cochliomyia hominivorax*, *Dendrolimus punctatus*) were significantly correlated with meteorological factors and its host plants (Fresia et al., 2013; Li et al., 2019). Climatic and habitat changes induced by multiple ice age cycles during the Pleistocene in Europe must have had an impact on the distribution of the grasshopper *Oedaleus decorus* (Kindler et al., 2012).

Given that the fossil record for *D. japonica* is unknown, phylogeographic methods based on divergence rates of genetic loci were used to date the lineage divergences and investigate possible signs of demographic events. Small population numbers, uneven sampling, and a single genetic marker‐based analysis hampered the interpretation of the results. However, genome‐wide single nucleotide polymorphisms (SNPs) and larger population sample sizes could potentially reveal more fine‐scaled patterns. In summary, our results indicated that *D. japonica* in mainland China remained almost stable over long periods of time. Although our present study provides some insight for the *D. japonica* genetic patterns, the study for *D. japonica* does not stop here. Further phylogeographic studies based on more extensive sampling would be needed to identify specific locations of glacial refugia.

## CONFLICT OF INTERESTS

The authors declare that the research was conducted in the absence of any commercial or financial relationships that could be construed as potential competing interests.

## AUTHOR CONTRIBUTIONS

ZZJ: Conception of the ideas. ZYX, GB, ML, and WWJ: Conduction of the fieldwork and collection of the data. ZZJ, ZYX, and GB: Analysis of the data. ZZJ and ZYX: Preparation of the manuscript.

## Supporting information

Fig S1

Table S1

Table S2

## Data Availability

Mitochondrial COI‐5P haplotype sequences are available at GenBank (MT678990‐MT679148). Microsatellite genotypes are available in the Dryad Digital Repository (https://doi.org/10.5061/dryad.z34tmpgbg ).
